# Basic research on antitoxin profiles: a key link to the management of snakebites

**DOI:** 10.2478/abm-2025-0033

**Published:** 2025-12-31

Snakebites are one of the World Health Organization’s neglected tropical diseases (NTDs) that are categorized as a global public health problem [1]. Outcomes of the treatment for snakebite can range from death to amputations or full recovery, depending on community factors (first aid care and timely patient transportation) and the health care system (antivenom accessibility and the availability of competent healthcare providers) [2]. Many unwanted outcomes can be prevented through the use of appropriate first aid methods, timely arrival at healthcare facilities, and proper access to specific types of antivenoms. Snake venoms prominently include hemotoxic/cytotoxic (extensive swelling, internal hemorrhage, and tissue necrosis), myotoxic (muscle breakdown and nephropathy), and neurotoxic (muscle paralysis) subtypes [2–5]. Interestingly, the neurotoxic and myotoxic venoms of *Daboia siamensis* (Eastern Russell’s viper), a common Russell’s viper in Asia, especially in Thailand [5] and China [6], contain the phospholipase A_2_ (PLA_2_) enzyme that damages cell membranes through the hydrolysis of phospholipids into fatty acids and lysophospholipids, leading to bleeding and motor nerve terminal damage, especially with the delayed antivenom administration [7]. Although the PLA_2_ enzyme in *D. siamensis* venom is highly complex and exhibits geographical variation, PLA_2_ is one of the most abundant and toxic components in snake venom, causing tissue damage, myotoxicity, and bleeding. Neutralization of the PLA_2_ enzyme is therefore a possible crucial therapeutic strategy.

Of interest, certain snakes can provide immunity to the venoms of other snakes. In this regard, PLA_2_ inhibitors are identified in the blood and internal organs of several snakes. For example, the gamma-type of PLA_2_ inhibitor (PLIγ) is identified from the serum of the non-venomous ringed water snake (*Sinonatrix annularis*) [8]. In this issue, Thaveekarn et al. [9] demonstrated PLA_2_ activity in five various venomous snakes, with the highest PLA_2_ activity in *D. siamensis*. The sequencing of PLIγ from *S. annularis* was used to synthesize a PLIγ that attenuates the hemorrhagic activity of *D. siamensis* venom in mice, as indicated by the minimum hemorrhagic dose. Additionally, PLIγ neutralizes PLA_2_ activity of crude *D. siamensis* venom by up to 34.8% in *in vitro* and decreases hemorrhagic spots on the inner surface of mouse skin by approximately 30.2% compared with controls. The authors concluded that PLIγ effectively inhibited PLA_2_ activity and mitigated the hemorrhagic effects induced by *D. siamensis* venom [9]. The findings on PLIγ contribute to the broader research initiative aimed at developing next-generation antivenoms that do not neutralize the venom based on immune responses, but rather utilize the enzymatic function. However, the enzymatic venom neutralization needs further refinement to produce antivenoms with improved safety and efficacy profiles. Currently available commercial anti-snake venoms are often associated with adverse allergic reactions (serum sickness) and exhibit limited effectiveness against PLA_2_ toxins. The combination of immunity- and enzymatic-based anti-snake venom might reduce the side effects and improve anti-venom effectiveness, especially through the reduction in PLA_2_-induced tissue damage. More studies are crucially required.

Finally, basic hospitals can appropriately manage snakebite subjects when the suitable anti-venoms are at hand with the proper selection of the appropriate anti-venoms. This is essential if envenomed patients are to be treated early. Basic research on antitoxin profiles is still essential for all health facilities to successfully care for their snakebite subjects [10].
